# Reducing Stigma in Lung Cancer Screening: Co‐Design of a Targeted Resource for Health Professionals in Australia

**DOI:** 10.1111/hex.70571

**Published:** 2026-04-27

**Authors:** Kathleen McFadden, Nathan J. Harrison, Shiho Rose, Giselle Hollinshead, Nicole M. Rankin, Marianne Weber, Kate Dunlop, Dan Luo, Brooke Nickel, Nehmat Houssami, Rachael H. Dodd

**Affiliations:** ^1^ The Daffodil Centre, The University of Sydney, and Cancer Council NSW Sydney Australia; ^2^ Flinders Health and Medical Research Institute, College of Medicine and Public Health, Flinders University Adelaide Australia; ^3^ NHMRC Centre of Research Excellence on Achieving the Tobacco Endgame, School of Public Health, The University of Queensland Herston Queensland Australia; ^4^ Consumer Advocate/Advisor Sydney Australia; ^5^ Melbourne School of Population and Global Health, Faculty of Medicine, Dentistry and Health Sciences, The University of Melbourne Melbourne Australia; ^6^ Sydney School of Public Health, Faculty of Medicine and Health, The University of Sydney Sydney Australia

**Keywords:** co‐design, implementation, lung cancer screening, preventive health, smoking, stigma

## Abstract

**Introduction:**

Organised lung cancer screening (LCS) programs are being developed worldwide in response to recent landmark trial evidence. Targeting high‐risk individuals primarily based on smoking history, LCS faces challenges from smoking‐related stigma, which can cause psychological harm and hinder participation. With limited interventions addressing this issue, this study aimed to co‐design a communication‐based strategy to reduce stigma in the LCS context.

**Methods:**

Using best‐practice co‐design principles, this study involved workshops, focus groups, and interviews with 5 consumers (people eligible for LCS in Australia) and 44 health professionals and other experts (including clinicians, radiographers, behavioural scientists, and health managers). Participants were purposively sampled to achieve representation across key variables – e.g., smoking history for consumers, professional roles for experts. Consultation with experts was conducted as part of a larger project developing a suite of information materials for the National Lung Cancer Screening Program in Australia. Key literature and concurrent qualitative research with consumers (*n* = 24) also informed co‐design. Qualitative data were analysed using abductive thematic analysis.

**Results:**

Early consultation identified the preferred format for the strategy as a resource targeted for health professionals involved in the LCS pathway. Resource content was iteratively revised, with three key themes developed: (1) Taking the onus off the individual; (2) Fostering understanding and empathy; (3) Positive framing. A lexicon guide promoting person‐first and empowerment language was also included guided by previous literature. The final resource was a four‐page A4 document for health professionals and LCS staff, designed to support effective, stigma‐sensitive communication during LCS.

**Conclusion:**

We co‐designed a practical, scalable resource to reduce stigma for use in the Australian National Lung Cancer Screening Program, expected to ultimately reduce psychological harm for LCS participants and enhance screening participation. Future research should evaluate the resource as an implementation strategy for LCS uptake. Cultural adaptation and tailoring of the resource for specific populations considering intersectional stigmas is also needed.

**Patient or Public Contribution:**

We collaborated with a consumer advisor for the project's duration, including study design, co‐facilitation of consumer workshops, analysis and manuscript writing.

## 1 Introduction

Screening for lung cancer with regular low‐dose computed tomography (CT) scans is effective for reducing lung cancer mortality. This has been demonstrated in two large‐scale randomised trials (the most recent published in 2020) [1, 2], with growing supporting evidence from smaller pilot or jurisdictional studies [3]. As a result, active design of lung cancer screening (LCS) programs is underway globally. To ensure that only those at the highest risk are screened, LCS is targeted based on the number one risk factor for lung cancer: smoking. Unlike population‐based cancer screening programs, which base eligibility criteria primarily on age alone, this means that LCS has unique psychosocial complexities associated with smoking, including smoking‐related stigma. Post‐diagnosis, stigma in lung cancer is widespread [4, 5]; however, less is known about how it impacts people pre‐diagnosis (such as during screening). Initial evidence suggests that stigma during LCS can (1) cause psychosocial harm to participants, and (2) undermine screening engagement [6]. The latter is of particular importance given that ongoing adherence to LCS (i.e., attendance for repeat scans) is instrumental in achieving the required clinical effectiveness outcomes to make screening worthwhile [7].

Few interventions to reduce or manage stigma in LCS specifically have been published. Williams et al. [8] tested an education‐based intervention that included lung cancer stigma‐minimising content with underserved groups eligible for screening. The reported reductions in perceived stigma were encouraging, though the drivers and mechanisms behind these results were unclear, as it was part of a broader educational intervention. A randomised controlled trial (RCT) of an invitation leaflet designed to target various psychosocial barriers to LCS, including stigma, found no difference in uptake of screening, though similarly, the impact of stigma‐related messaging alone was not discernible [9]. Outside of LCS, a nurse‐led behavioural intervention aimed at encouraging earlier presentation to primary care for those at higher risk of lung cancer has shown promise for reducing stigma (among multiple other barriers) and improving medical help‐seeking [10, 11]. The intervention has been described as non‐judgemental, avoided ‘lecturing’ and was conducted in a non‐threatening and relaxed environment [10].

The potential for tailored decision aids and targeted empathetic messages to reduce stigma in LCS has also been raised [5]. For those diagnosed with lung cancer or chronic obstructive pulmonary disease (COPD), a recent systematic review found a range of promising stigma‐reduction interventions (including educational, behaviour change‐focused, and psychotherapeutic strategies) [12], though most were in pilot or feasibility stages and required additional evaluation. Interventions showing the most success were those based on established psychosocial therapy techniques, such as cognitive behavioural therapy or acceptance‐commitment therapy, and implemented longer term (6 weeks to 6 months) [12]. Such resource‐intensive interventions may not be feasible for the high participant volumes seen in LCS. Wide‐reaching public campaigns, such as those by Lung Foundation Australia and similar international organisations [13, 14], may be effective for reducing societal‐level stigma [13, 14]. Following two campaigns conducted by Lung Foundation Australia reaching almost 10% of the Australian population in 2019, 54% of those recalling the campaigns reported greater empathy towards people with lung cancer as a result [14]. However, no public campaigns related to LCS, or about those eligible for LCS, have been tested.

In other behaviour‐based health stigma contexts (e.g., obesity, addiction), types of messaging (e.g., narrative‐based or personal stories) combined with acknowledgement of factors outside of an individual's control may reduce stigma [15, 16, 17]. However, these techniques and themes have not been tested in the smoking or lung cancer context.

Multiple language guides and communication assessment tools have been created to support reducing stigma associated with lung cancer and smoking [18, 19, 20]. In particular, the recently published Lung Cancer Stigma Communications Assessment Tool (LCS‐CAT) assists in auditing and replacing content to minimise language, imagery, or context that may be stigmatising [20]. These tools provide a low‐cost, scalable way to reduce potential stigma in general communication materials. They may also provide foundational themes and content for the development of targeted stigma‐reducing interventions.

Considering (1) the growing number of LCS programs internationally, (2) the acknowledged burden for participants and risk to program efficacy that stigma presents, and (3) the lack of stigma‐reduction strategies for LCS, this study was conceived to address a critical need. In tandem with the development of key messages and information materials for the Australian National Lung Cancer Screening Program (NLCSP), the goal of this study was to co‐design a strategy to be used alongside implementation of the program, to reduce stigma in LCS. For clarity and consistency, we refer to the co‐designed end product throughout this manuscript as the ‘resource’, rather than the broader terminology of ‘strategy’ or ‘solution’ commonly used in co‐design frameworks. However, the resource format was not pre‐determined and was derived during the co‐design process and consultation.

## 2 Materials and Methods

### 2.1 Methodology Overview

The aim of co‐design is to meaningfully engage end users in health research, to improve processes, efficiency and health outcomes [21, 22]. Our use of the term ‘co‐design’ includes both consultation and co‐development of the research itself (via collaboration with a consumer advisor) and active engagement with end users in developing solutions to a predetermined problem [22]. This study was guided by the principles and approach for co‐design from the NSW Health Agency for Clinical Innovation (ACI) [23]. An overview of the activities in this project, aligning to the ACI co‐design toolkit process, is presented in Table 1. As co‐design is purposively dynamic and iterative, we were not prescriptive in the exact procedures (e.g., number of workshops/iterations of strategies). Instead, progress was regularly assessed by the study team and procedures were adapted accordingly.

**Table 1:** Project co‐design process aligning to the NSW Health Agency for Clinical Innovation co‐design toolkit [23].

Step of ACI co‐design model	Activities/process
Start‐up and engage Frame the project problem, build the team and get ready for co‐design	Engagement & partnering with a consumer advisor to support study design and conduct.
Gather Build understanding by learning from lived experience	Explore the problem of stigma in lung cancer screening by engaging with: people eligible for lung cancer screening in Australia, with a smoking history and potentially lived experience of stigma in healthcare (consumers) health professionals and other relevant experts (experts) published literature
Understand Make sense of what you have learnt; identifying the key touchpoints and opportunities to improve	Summarise findings from ‘Gather’ step, and identify key insights and themes Identify opportunities for strategies* to reduce or manage stigma in lung cancer screening
Improve Create solutions; test and learn; adapt; implement and evaluate	Create/mock‐up an initial design of the resource Get feedback, test and build on/revise the resource

* As part of the co‐design process, the format of the ‘strategy’ for reducing stigma was determined as a resource targeted for health professionals.

### 2.2 Study Team

The research team included those with previous experience and expertise in cancer screening, lung cancer stigma, smoking cessation and participatory methodologies. During initial study conception, we engaged a consumer advisor (GH) as part of the study team. GH has experience in research engagement from idea development through to thematic analysis from a consumer perspective, and has undertaken relevant training in consumer engagement in research from Cancer Council NSW and Health Consumers NSW. GH contributed throughout the study – including in design and methodology (before Ethics submission); developing workshop content; co‐facilitation of workshops; discussion of field notes and key ideas/themes arising from workshops.

### 2.3 Participants and Recruitment

Both consumers and health professionals/other relevant experts were included in the study, as they would be the end users of a strategy to reduce stigma. Consumers approached were those considered eligible for LCS in Australia, that is, a person aged 50–70 years with a history of tobacco cigarette smoking of at least 30 pack‐years, and, if no longer smoking, had quit within the previous 10 years [24]. Health professionals and experts included general practitioners, primary care practice managers, medical and nursing specialists (respiratory medicine and other), radiologists and radiographers, pharmacists, behavioural scientists and health managers.

Consumers were recruited using (1) targeted emailing from contact lists of relevant participant networks (e.g., Lung Foundation Australia); (2) public advertisement of the study by local health networks or consumer groups; (3) existing research relationships with consumers; and (4) snowballing. Consumers were only directly contacted if permission was granted by data managers or custodians, or if consumers had indicated in previous relevant studies they would like to be invited for future research.

Health professionals and experts were recruited as part of another study focused on designing the information materials and baseline key messages for the Australian NLCSP [25, 26]. We used similar recruitment methods as for consumers, in addition to targeted emailing of those known to the research team. In recruiting health professionals, we sought a diversity of job roles and experience – including those with expertise and those naïve to LCS. As the Australian NLCSP is primary care‐led, we aimed for approximately half of consultations to be with primary care staff.

Interested participants were given all study information and a link to an Expression of Interest (EOI) Form (including demographic questions), Participant Information Sheet and Consent Form hosted by The University of Sydney in Qualtrics. Once the EOI and signed consent form were completed and received, participants were contacted about consultation activities.

### 2.4 Data Collection – Co‐Design Process

**Figure 1:**
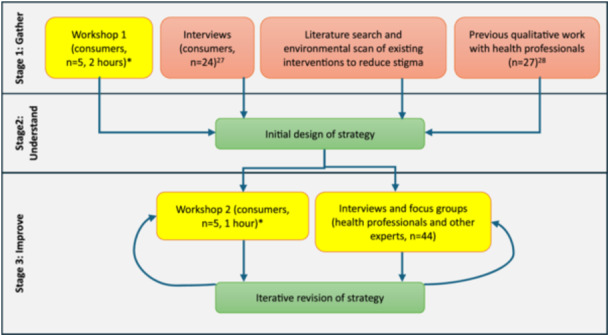
Co‐design process and activities involved in the development of the resource. *co‐facilitated with consumer advisor (GH). Yellow boxes indicate consultation activities undertaken specifically as part of the co‐design study.

An overview of the co‐design process and activities is presented in Figure 1. The initial design of the resource was informed primarily by a purpose‐built workshop with consumers (Workshop 1; content and format described in Appendix C). Other inputs included: existing literature on effective interventions to reduce stigma in healthcare, and previous qualitative work conducted by the research team with (a) consumers about views, expectations and potential impacts of stigma (conducted in tandem with this project) [27], and (b) LCS health professionals exploring psychosocial outcomes associated with LCS (where smoking‐related stigma was a major theme [28]).

Following the initial design, the resource was iteratively revised, including via a second purpose‐built workshop with consumers (Workshop 2; content and format described in Appendix C), and interviews and focus groups with health professionals and experts. The latter were conducted as part of consultations about a broader suite of information materials designed as part of the Australian NLCSP. Following consultation, all experts were invited to provide additional written feedback or mark‐ups of the resource via email. In one case, a participant could not attend an interview or workshop and so provided written feedback only. At all stages, input was also sought from the research team and other experts informally.

The workshops with consumers were purpose‐built for this co‐design project. They involved a mixture of structured presentation, group discussion and activities (full structure and content is presented in Appendix C). Workshops were facilitated by KM (lead investigator) and GH (consumer advisor), with RD (senior researcher) also in attendance. Consumers were offered a $50 gift card per hour of participation.

For consultations with health professionals and other experts, information was sent in advance to participants to allow time for pre‐perusal. There was no specific interview guide; during interviews and focus groups, information was shared on the screen and worked through with the facilitator (KM, RD, MW, KD and DL). Health professionals and experts gave feedback on the content and design and were asked about the usability in practice; they were offered a $100 gift card per hour of participation.

Interviews and workshops were conducted (where possible) via Zoom and in some cases via telephone. All consultations were audio recorded and transcribed using The University of Sydney's Microsoft Word license. Transcripts were de‐identified by removing participants' names and any other identifying information. Field notes were taken during consumer workshops by KM.

### 2.5 Data Analysis

Following all workshops and interviews, initial data familiarisation was completed by one investigator (KM), involving re‐reading transcripts, making preliminary notes and identifying potential themes and patterns. After consumer workshops, field notes were also discussed among workshop facilitators and attendees (KM, RD, GH). We used abductive thematic analysis to interpret data, as this allows for both theory‐driven (top‐down, to frame using existing models and understanding of health‐related stigma [29]) and data‐driven (bottom‐up, generation of new ideas) themes [30, 31]. Analysis was completed primarily in Excel, with data interpretation discussed among the study team following consultation activities. Themes and sub‐themes formed the content of the resource, and as the document took form, it was iteratively updated in Adobe Acrobat as a PDF. Following the ‘Gather’ and ‘Understand’ stages, participants in consultation during the ‘Improve’ stage were presented with a mock‐up of the resource with initial themes and content and asked to provide feedback. Final themes and subthemes were developed by the lead investigator following discussion and consensus with workshop facilitators (KM, RD and GH).

### 2.6 Ethical Considerations

Ethics approval for all consultation stages was obtained from The University of Sydney Human Research Ethics Committee (2023/HE000867, 2024/HE000248, 2024/HE000700).

## 3 Results

### 3.1 Participant Characteristics

Characteristics of consumer and health professional/expert participants are described in Table 2. Five consumers took part in both workshops. The mean age of consumers was 59 years (ranging from 53 to 66) and 60% (*n* = 3) identified as female. Three consumers (60%) reported that they were currently smoking, and two (40%) had quit within the past 10 years. All consumers lived in major cities and had self‐reported high levels of health literacy [33]; all except one had studied after high school.

**Table 2:** Participant characteristics – consumers and health professionals/other experts.

Consumers (*n* = 5)	No. of participants
Age (Mean, Range)	59 (53–66)
Gender (Female)	3
Identify as culturally and linguistically diverse	2
Highest level of education	
School certificate or intermediate certificate (or equivalent)	1
Diploma or certificate	2
University degree	2
Relationship status	
Married, or living with partner	2
Divorced or separated	3
Confidence in filling out medical forms without help	
Extremely	3
Quite a bit	2
Smoking status	
I am currently someone who smokes	3
I am someone who has quit smoking in the last 10 years	2
Previous cancer diagnosis	
Yes (other type of cancer)	2
No	2
Don't know	1
Previous cancer diagnosis – immediate family (parents, siblings, children)	
Yes (lung cancer)	1
Yes (other cancer)	4
Self‐reported general health	
Good	2
Fair	3
Remoteness [32]	
Major Cities	5
**Health professionals and other relevant experts (*n*** = **44)**	
**Role**	
Primary care	22 (50%)
General Practitioner	21 (48%)
Practice Manager	1 (2%)
Lung cancer clinicians/specialist	7 (16%)
Lung cancer or oncology nurse	2 (5%)
Respiratory physician	3 (7%)
Medical oncologist	1 (2%)
Surgeon	1 (2%)
Pharmacist	1 (2%)
Health promotion, screening or smoking cessation manager/expert	11 (25%)
Radiographer	2 (5%)
Radiologist	1 (2%)
**State**	
Australian Capital Territory	4 (9%)
New South Wales	19 (43%)
Northern Territory	1 (2%)
Queensland	3 (7%)
South Australia	1 (2%)
Tasmania	2 (5%)
Victoria	9 (21%)
Western Australia	5 (11%)

In total, 44 health professionals/experts contributed to the iterative design of the resource, participating in either an individual interview (*n* = 15), as part of a focus group (7 focus groups, *n* = 28) or providing written feedback only (*n* = 1). In total, 50% (*n* = 22) worked in primary care, 16% (*n* = 7) in lung cancer specialties and 25% (*n* = 11) in health promotion, screening or smoking cessation behavioural science roles. Most participants (43%, *n* = 19) were from New South Wales, though all states and territories were represented.

### 3.2 Resource Development

Results are presented by the type of information (e.g., format or content of resource), rather than by stage of the co‐design process.

#### 3.2.1 Format of Strategy (Resource)

A key theme that was raised in all contributing inputs to initial resource development (Stage 1 in Figure 1) was that perceived or experienced stigma from health professionals is a central barrier to screening. People who smoke or have a smoking history reported often feeling sidelined, not believed, forgotten about or openly discriminated against in the health system.

The research team had initially envisaged that the designed strategy would target intrapersonal (self) stigma based on existing successful interventions in other contexts. However, in workshops and interviews, consumers believed that stigma in LCS would primarily be driven by the interactions that people have with health professionals and those delivering LCS (GPs, radiographers, receptionists, nurses, practice managers) along the screening and assessment pathway. This aligns with evidence suggesting that some health professionals hold implicit stigmatising views towards people with lung cancer and people who smoke [34, 35]. Through Workshop 1 and discussion with the research team, the optimal ‘strategy’ was therefore determined as a resource targeted to health professionals aimed specifically at reducing stigma and improving communication practices in LCS.

The LCS screening and assessment pathway includes touchpoints from recruitment through to results delivery and ongoing participation in LDCT scans over time. Stigma felt from health professionals or those delivering LCS can occur at any stage of the pathway. Therefore, the resource was designed to support health professionals' initial familiarisation with the program and serve as an ongoing reference as part of a broader collection of information materials available to those delivering the Australian NLCSP. As part of a concurrent project developing the suite of information materials for the NLCSP [36], this resource was then co‐designed during further consultation activities (Stages 2 and 3 in Figure 1). Consultation focused on developing the content and wording for the resource, with input from the Australian Government (Cancer Australia, the Department of Health, Disability and Ageing), and the National Aboriginal Community Controlled Health Organisation (NACCHO). This was then styled, formatted and professionally designed in accordance with brand guidelines for the NLCSP materials.

#### 3.2.2 Thematic Content

**Figure 2:**
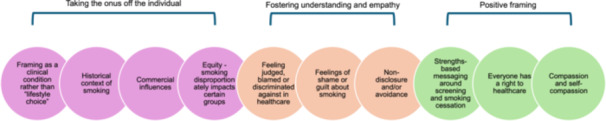
Key content themes included in the resource.

Three key themes were developed during analysis of consultation content, as well as consideration of other inputs in iterative development of the resource. Themes and sub‐themes are described below and displayed in Figure 2. Exemplar quotes representing each sub‐theme are presented in Table 3.

**Table 3:** Exemplar quotes for each theme.

Theme	Subtheme	Exemplar quote
Theme 1: Taking the onus off the individual	Theme 1.1: Framing as a clinical condition rather than ‘lifestyle choice’	People just don't understand addiction and how it affects people, how you can't stop. The same with addiction for gambling, alcohol … there isn't a great deal of understanding or acceptance. – Consumer [commenting on the resource] I think it's a good forward reminder about considering the language that we use and framing it not as a personal deficit because you choose to smoke… because often when we talk clinically, it tends to sneak into the language. – Health professional/Expert
	Theme 1.2: Historical context of smoking	The people of today don't realise … what smoking was back when we started doing it. – Consumer If it was invented now, it would never be legalised. – Consumer We're talking about a generational thing here … we weren't educated like [young medical practitioners] were. We were subject to just a bunch of footballers who used to have a smoke at half time … morning tea was called ‘Smoko’ and still is in a lot of cases. – Consumer
	Theme 1.3: Commercial influences	Many of us were exposed to those glamorised and glorified advertisements and those pseudo‐health‐based advertisements for smoking. So, ‘if you're stressed, have a smoke’ … ‘if you want to stay thin, have a smoke’. – Consumer [written comment on resource] Can we mention industry here? That the tobacco industry knew and designed cigarette to be addictive – this was deliberate. – Health professional/Expert
	Theme 1.4: Equity	We are talking about equity, and we need it for the LGBTQIA [community] – [they have] high rates of smoking … and greatest stigma. – Health professional/Expert
Theme 2: Fostering understanding and empathy	Theme 2.1: Internalised stigma	You feel like you're stopping somebody with lung cancer or cervical cancer from getting their treatment because you're clogging up the works when you shouldn't have smoked. – Consumer
	Theme 2.2: Externalised stigma	Some medical practitioners do essentially become dismissive of you … or they can be dismissive of your conditions … this is another opportunity to make the point that discrimination has probably been experienced. – Consumer
	Theme 2.3: Non‐disclosure and avoidance of screening/other healthcare	I'm pretty sure I've lied about my smoking at a medical practitioners’. – Consumer The emotional stigma side of it might stop somebody from moving forward in getting help. It's been a bit of an issue for me. – Consumer Remind people that the screening is confidential. – Consumer
Theme 3: Positive framing	Theme 3.1: Strengths‐based messaging around screening and smoking cessation	The one that I found really positive is ‘never quit quitting’. And I thought, ‘Well, there you go! That's a positive way of putting it’. That actually made me feel encouraged … because that's what happens when you try to quit, you fail, and you go back and [try again]. – Consumer So if they could do some positive stories where screening has saved people's lives that will help other people. – Consumer
	Theme 3.2: Everyone has the right to healthcare	[LCS must be] non‐judgmental. Regardless of whether they smoked or not, no one deserves lung cancer. – Consumer Everyone deserves to be treated with respect and supported in their health care. – Consumer
	Theme 3.3: Compassion and self‐compassion	Just because I found it easy to give up… [doesn't mean] someone else does. – Consumer Remind people to be kind with themselves. – Consumer

##### 3.2.2.1 Theme 1: Taking the Onus Off the Individual

###### 3.2.2.1.1 *T*heme 1.1: Framing smoking as a clinical condition rather than ‘lifestyle choice’

The harmful narrative of personal responsibility associated with smoking was raised consistently by consumers and experts during consultations. All participants felt that smoking is generally seen as a modifiable behaviour or habit, and rarely contextualised clinically as a nicotine dependence or addiction. As a result, participants expressed that continued smoking is seen as a lack of motivation or determination to stop. Notably, some experts flagged that framing tobacco smoking as an ‘addiction’ may add unintended stigma associated with having an addiction. This aligns with some dichotomy in the literature about the benefits and harms of using the brain disease model of addiction in conceptualisation of tobacco use.

###### 3.2.2.1.2 Theme 1.2: Historical Context of Smoking

Participants consistently cited external determinants of smoking being poorly understood or overlooked as a key contributor to stigma. Specifically, both consumer and health professionals/expert participants flagged that when people eligible for LCS started smoking, there was (a) entrenched normalisation of smoking in society; and (b) a lack of research, education or tobacco control measures that are available and ubiquitous today.

###### 3.2.2.1.3 Theme 1.3: Commercial Influences

All participants flagged that the role of commercial interests in driving tobacco use are not well‐recognised. Many mentioned that cigarettes used to be widely advertised (marketed as ‘cool’, ‘glamorous’ or even having health benefits). Others pointed out the complex political and economic context around the sale of cigarettes (‘why are they still legal?’), and that focusing on smoking as an individual's responsibility is a profit‐driven tactic of the tobacco industry.

###### 3.2.2.1.4 Theme 1.4: Equity

Participants – specifically experts – identified that smoking, and its harms, disproportionately impact certain groups (e.g., Aboriginal and Torres Strait Islander peoples, with some reporting this as an ongoing impact of colonisation). Additionally, many participants said that there are various reasons for starting and continuing smoking, and these can be inherently linked to privilege and equity. For example, most consumers flagged that smoking is often used as a ‘crutch’ for other personal issues (e.g., socioeconomic pressures, mental health issues), so for some people, stopping or reducing smoking may not be, or seem like, an option.

##### 3.2.2.2 Theme 2: Fostering Understanding and Empathy

Many participants expressed that health professionals may benefit from information about how people who have a smoking history feel, or expect to feel, during healthcare encounters. Points specifically related to internalised and externalised stigma, and the outcomes of these, were raised. Many noted the importance of not laying blame on health professionals, but only reminding those delivering LCS how people who smoke feel.

###### 3.2.2.2.1 Theme 2.1: Internalised Stigma

Feelings of shame, guilt and embarrassment about smoking were commonly reported in healthcare situations by consumer participants. As a result of the personal responsibility narrative around smoking, some reported that they should live with the consequences of smoking or are undeserving of healthcare and acknowledged that both aspects may act as a barrier to screening.

###### 3.2.2.2.2 Theme 2.2: Externalised Stigma

Consumers often reported feeling judged or blamed in healthcare encounters – this may not be enacted but could be perceived or anticipated as a result of previous stigmatising experiences. Consumers also reported previously feeling ignored, brushed off, or that ‘everything is brought back to smoking’. Fear of judgement and an expectation to be dismissed or not listened to were both identified as barriers to screening.

###### 3.2.2.2.3 Theme 2.3: Non‐Disclosure and Avoidance of Screening/Other Healthcare

In addition to non‐engagement in LCS because of internalised or externalised stigma, non‐disclosure of smoking history was also flagged as a potential negative outcome of stigma. Consumers raised concerns that people may find talking about the eligibility criteria for LCS uncomfortable. It was also noted that people eligible for LCS may be reluctant to openly admit or discuss details or underreport smoking information. Consumers suggested that as public awareness of the NLCSP grows and people become more familiar with the eligibility criteria, participation in the program may inadvertently disclose their smoking history.

##### 3.2.2.3 Theme 3: Positive Framing

###### 3.2.2.3.1 Theme 3.1: Strengths‐Based Messaging Around Screening and Smoking Cessation

The value of strengths‐based messaging in discussions was perceived by all participants. This included both for LCS (e.g., the benefits of early detection for lung cancer outcomes) and for smoking cessation (e.g., stopping or reducing tobacco use at any time is beneficial for health). Additionally, reassurance that participation in LCS was not contingent on smoking cessation.

###### 3.2.2.3.2 Theme 3.2: Everyone Has the Right to Healthcare

The self‐infliction narrative associated with lung cancer and smoking was raised regularly, and consumers noted that people with a smoking history may feel like screening will be stigmatised for this reason. Reframing this stigmatising rhetoric to include and acknowledge everyone's universal right to healthcare was seen as effective. Some consumers also flagged that introduction of a targeted screening program for people with a smoking history made them feel ‘brought into the fold’, supporting the position that everyone has a right to healthcare.

###### 3.2.2.3.3 Theme 3.3: Compassion and Self‐Compassion

Aligning with feelings of inadequacy associated with not being able to quit smoking, participants flagged that compassion and self‐compassion were imperative for smoking cessation. This included messaging that people are not perfect, and negative feelings as part of smoking cessation are experienced by many. In addition, consumers found messaging related to ‘never quit quitting’ as particularly supportive, i.e., smoking cessation is a personal journey – it often takes many tries, and each attempt is a positive step towards understanding what might work for an individual.

### 3.3 Additional Content

Some health professionals identified that additional content specifically around use of language would be a useful inclusion in the resource. This is supported by recent literature and evidence‐based calls to action to use precision language and terminology, acknowledging that language has power and can intentionally or unintentionally reinforce stigma [18, 37, 38]. Previously published lexicon guides for lung cancer were consulted [20, 39, 40]; included terms were iteratively revised based on co‐design consultation for those most applicable and useful to the screening context (e.g., language alternatives related to treatment or cancer progression were excluded).

### 3.4 Final Co‐Designed Resource

Following ongoing consultation and refinement of thematic and additional content, the final iteration of the resource was a 4‐page A4 document to be included in the suite of information materials delivered to health professionals as part of the Australian NLCSP. The final resource is available online and has been sent to primary care practices physically with other resources about the NLCSP; the final resource is included in Appendix A and published online: www.health.gov.au/resources/publications/nlcsp-reducing-stigma. Mapping of key themes to the final resource is displayed in Appendix B.

## 4 Discussion

To the best of our knowledge, this study is the first co‐designed intervention or strategy to reduce smoking‐related stigma, and the first specifically for stigma in lung cancer screening. The content and format of the co‐designed resource was a culmination of interviews, focus groups and workshops with people eligible for LCS and health professionals/experts, as well as key literature. The final resource was a four‐page A4 document targeted for health professionals and those working in LCS (including GPs, radiographers, receptionists, nurses, practice managers) delivered as part of the Australian National Lung Cancer Screening Program.

The success of any LCS program relies on participation from appropriate priority groups. Given that stigma is a key barrier to LCS, this resource may be an effective implementation strategy for screening as a clinical intervention [41]. Alongside testing whether the resource directly reduces stigma and improves other psychosocial factors for LCS participants (or those eligible), measuring implementation outcomes associated with the resource is also a priority. Evidence for the feasibility and acceptability of the resource in practice is needed, as well as metrics on adoption for both consumers and health professionals [41, 42]. As a short resource available online (and printable) as part of the suite of information materials for health professionals for the Australian NLCSP, this intervention is expected to be scalable, implementable and cost‐effective. However, given that the NLCSP is new in Australia and facilitated by primary care, there is a need for education and training for primary care practitioners to become familiar with the program. Time required to read and assimilate new information has been cited as a key barrier for GPs in completing continuing medical education and implementing clinical guidelines in practice [43, 44]. There is a possibility that this resource may be deemed ‘non‐essential’ and, with time constraints, be sidelined in favour of practical or logistical information about LCS or the NLCSP. Improving broader acknowledgement and understanding of the deleterious effects of stigma on health outcomes may help to counteract this [4, 45].

Most stigma‐reduction interventions in the lung cancer and lung disease context are targeted at the patient or individual [12]. There are only a few designed specifically for health professionals [12], and a recent review highlighted the scarcity of interventions focused on healthcare workers for stigma reduction in cancer more generally [46]. Three studies reported results of a group‐based educational intervention for stigma reduction in lung cancer, finding that while professionals' self‐reported ability to communicate empathetically with lung cancer patients increased significantly following the intervention [47, 48, 49], patients' experiences of stigma were unchanged [47, 48]. In the evaluation of the current resource, this highlights the need to ensure any reductions in stigma are translated through to the patient experience, and that these outcomes are measured in the assessment of the intervention.

Findings from this study can also be used to inform the development of mass media and targeted communications campaigns for the Australian NLCSP, and other programs internationally. Specific thematic takeaways include using messaging that helps reverse harmful narratives around individual responsibility for smoking, that increases empathy and understanding about how people who smoke or with a smoking history experience healthcare, and that are strengths‐based. Use of this content may help to shift broader public sentiment around lung cancer and smoking, reducing stigma and improving outcomes for people at‐risk and diagnosed with respiratory conditions.

The key strengths of this study stemmed from the collaborative participatory methods. Co‐design with a range of stakeholders (both consumers and health professionals/experts) enabled a variety of perspectives to be considered in the development of the resource. Engagement with a relatively large sample of experts from across Australia and across many clinical roles was also critical for this resource, given it is targeted at health professionals and those involved in delivering LCS along the entire screening and assessment pathway. The sample of consumers was relatively small, and participants had high levels of education and self‐reported health literacy, which does not necessarily represent the diverse group of LCS‐eligible individuals in Australia (estimated 13%–14% of the Australian population; 930,000 eligible as at 1 July 2025) [50] While some participants did identify as having culturally and linguistically diverse backgrounds, stigma and its impacts differ across communities [6]. Intersectional stigma is a critical consideration in LCS, as characteristics associated with smoking (such as socioeconomic status, race, or mental health) may also carry separate stigmas [6, 51]. These overlapping stigmas can compound barriers to access, underscoring the need for tailored stigma‐reduction strategies for specific priority populations. As LCS currently stands, using smoking history as the primary eligibility criterion may also overlook how other identity facets coincide with smoking to create compounded risks and barriers that could affect screening effectiveness and equity. Purposeful design and adaptation for different cultural contexts is needed, and investment in tailoring resources should be prioritised in future research.

## 5 Conclusion

This project used a rigorous co‐design process to develop a practical, scalable resource to reduce stigma for implementation in Australia's National Lung Cancer Screening Program. Targeted for health professionals, the resource is expected to support LCS participation, ultimately reducing psychological harm to participants and improving screening engagement. Measurement of these outcomes and testing of the resource as an implementation strategy for LCS are avenues for further research.

## Author Contributions

**Nathan J. Harrison:** conceptualization, methodology, investigation, writing – original draft, writing – review and editing, formal analysis, validation. **Shiho Rose:** methodology, conceptualization, investigation, validation, formal analysis, writing – review and editing, writing – original draft. **Kate Dunlop:** data curation, writing – review and editing, investigation. **Dan Luo:** data curation, writing – review and editing, investigation. **Rachael H. Dodd:** conceptualization, methodology, data curation, supervision, investigation, validation, formal analysis, project administration, writing – review and editing, writing – original draft.

## Ethics Statement

Ethics approval for all consultation stages was obtained from The University of Sydney Human Research Ethics Committee (2023/HE000867, 2024/HE000248, 2024/HE000700).

## Conflicts of Interest

The authors declare no conflicts of interest.

## Supporting information

Appendices HEX jan.

## Data Availability

The authors have nothing to report.

## References

[1] H. J. de Koning , C. M. van der Aalst , P. A. de Jong , et al., “Reduced Lung‐Cancer Mortality With Volume Ct Screening in a Randomized Trial,” New England Journal of Medicine 382, no. 6 (2020): 503–513, 10.1056/NEJMOA1911793/SUPPL_FILE/NEJMOA1911793_DATA-SHARING.PDF. 31995683

[2] “Reduced Lung‐Cancer Mortality With Low‐Dose Computed Tomographic Screening,” New England Journal of Medicine 365, no. 5 (2011): 395–409, 10.1056/NEJMOA1102873. 21714641 PMC4356534

[3] S. J. Adams , E. Stone , D. R. Baldwin , R. Vliegenthart , P. Lee , and F. J. Fintelmann , “Lung Cancer Screening,” Lancet 401, no. 10374 (2023): 390–408, 10.1016/S0140-6736(22)01694-4/ASSET/F5650D19-794B-4DD8-B2AE-219B5BA3623F/MAIN.ASSETS/GR1.JPG. 36563698

[4] H. A. Hamann , T. J. Williamson , J. L. Studts , and J. S. Ostroff , “Lung Cancer Stigma Then and Now: Continued Challenges Amid a Landscape of Progress,” Journal of Thoracic Oncology 16, no. 1 (2021): 17–20, 10.1016/j.jtho.2020.10.017. 33384057 PMC8020298

[5] H. A. Hamann , E. S. Ver Hoeve , L. Carter‐Harris , J. L. Studts , and J. S. Ostroff , “Multilevel Opportunities to Address Lung Cancer Stigma Across the Cancer Control Continuum,” Journal of Thoracic Oncology 13, no. 8 (2018): 1062–1075, 10.1016/J.JTHO.2018.05.014. 29800746 PMC6417494

[6] S. Miriyala , K. V. Nguyen , A. Park , T. Hwang , M. C. Aldrich , and J. Richmond , “Racism, Discrimination, Medical Mistrust, Stigma, and Lung Cancer Screening: A Scoping Review,” Ethnicity & Health 30 (2025): 1–26, 10.1080/13557858.2025.2458303. 39901346 PMC11961322

[7] S. S. Han , S. A. Erdogan , I. Toumazis , A. Leung , and S. K. Plevritis , “Evaluating the Impact of Varied Compliance to Lung Cancer Screening Recommendations Using a Microsimulation Model,” Cancer causes & control: CCC 28, no. 9 (2017): 947–958, 10.1007/S10552-017-0907-X/FIGURES/4. 28702814 PMC5880208

[8] L. B. Williams , B. J. Shelton , M. L. Gomez , Y. D. Al‐Mrayat , and J. L. Studts , “Using Implementation Science to Disseminate a Lung Cancer Screening Education Intervention Through Community Health Workers,” Journal of Community Health 46, no. 1 (2021): 165–173, 10.1007/S10900-020-00864-2. 32594413 PMC8183677

[9] S. L. Quaife , M. Ruparel , J. L. Dickson , et al., “Lung Screen Uptake Trial (Lsut): Randomized Controlled Clinical Trial Testing Targeted Invitation Materials,” American Journal of Respiratory and Critical Care Medicine 201, no. 8 (2020): 965–975, 10.1164/RCCM.201905-0946OC/SUPPL_FILE/DISCLOSURES.PDF. 31825647 PMC7159423

[10] S. R. Murray , Y. Kutzer , E. Habgood , et al., “Reducing Barriers to Consulting a General Practitioner in Patients at Increased Risk of Lung Cancer: A Qualitative Evaluation of the Chest Australia Intervention,” Family Practice 34, no. 6 (2017): 740–746, 10.1093/FAMPRA/CMX057. 29155969

[11] S. R. Murray , P. Murchie , N. Campbell , et al., “Protocol for the Chest Australia Trial: A Phase Ii Randomised Controlled Trial of An Intervention to Reduce Time‐To‐Consult With Symptoms of Lung Cancer,” BMJ Open 5, no. 5 (2015): e008046, 10.1136/BMJOPEN-2015-008046. PMC4442170 25986641

[12] J. Yamazaki‐Tan , N. J. Harrison , H. Marshall , C. Gartner , C. E. Runge , and K. Morphett , “Interventions to Reduce Lung Cancer and Copd‐Related Stigma: A Systematic Review,” Annals of Behavioral Medicine 58, no. 11 (2024): 729–740, 10.1093/ABM/KAAE048. 39197098 PMC11487577

[13] Lung Health Foundation LCC . Start Asking the Right Questions About Lung Cancer Second Edition.; 2020, accessed May 1, 2025, https://thewrongquestion.ca/wp-content/uploads/2021/10/Start-Asking-the-Right-Question-About-Lung-Cancer-Second-Edition.pdf.

[14] Lung Foundation Australia. Australians' lung cancer understanding and attitudes: Summary of survey findings ‐ Lung Foundation Australia. 2020, accessed April 9, 2025, https://lungfoundation.com.au/news/australians-lung-cancer-understanding-and-attitudes-summary-of-survey-findings.

[15] J. Niederdeppe , S. Roh , and M. A. Shapiro , “Acknowledging Individual Responsibility While Emphasizing Social Determinants in Narratives to Promote Obesity‐Reducing Public Policy: A Randomized Experiment,” PLoS One 10, no. 2 (2015): e0117565, 10.1371/JOURNAL.PONE.0117565. 25706743 PMC4338108

[16] K. Heley , A. Kennedy‐Hendricks , J. Niederdeppe , and C. L. Barry , “Reducing Health‐Related Stigma Through Narrative Messages,” Health Communication 35, no. 7 (2020): 849–860, 10.1080/10410236.2019.1598614. 31014112

[17] E. McGinty , B. Pescosolido , A. Kennedy‐Hendricks , and C. L. Barry , “Communication Strategies to Counter Stigma and Improve Mental Illness and Substance Use Disorder Policy,” Psychiatric Services 69, no. 2 (2018): 136–146, 10.1176/APPI.PS.201700076. 28967320 PMC5794622

[18] International Association for the Study of Lung Cancer. *IASLC Language Guide*.; 2021, accessed December 5, 2024, https://www.iaslc.org/file/6879/download?token=XcWsYmS4.

[19] C. Lockstadt , M. M. Pasquinelli , J. Feldman , et al., “Brief Report: Precision Language and Deletion of the “S” Word,” JTO Clin Res Rep 6 (2024): 100711, 10.1016/j.jtocrr.2024.100711. 39758597 PMC11699356

[20] L. Carter‐Bawa , J. S. Ostroff , K. Hoover , and J. L. Studts , “Effective Communication About Lung Cancer Screening Without Iatrogenic Stigma: A Brief Report Case Study Using the Lung Cancer Stigma Communications Assessment Tool of Lungtalk,” JTO Clinical and Research Reports 4, no. 11 (2023): 100585, 10.1016/J.JTOCRR.2023.100585. 38029025 PMC10679887

[21] P. Slattery , A. K. Saeri , and P. Bragge , “Research Co‐Design in Health: A Rapid Overview of Reviews,” Health Research Policy and Systems 18, no. 1 (2020): 17, 10.1186/S12961-020-0528-9/TABLES/2. 32046728 PMC7014755

[22] C. Vargas , J. Whelan , J. Brimblecombe , and S. Allender , “Co‐Creation, Co‐Design, Co‐Production for Public Health ‐ a Perspective on Definition and Distinctions,” Public Health Research & Practice 32, no. 2 (2022): e3222211, 10.17061/PHRP3222211. 35702744

[23] NSW Health Agency for Clinical Innovation. Co‐design toolkit: Co‐design step‐by‐step, accessed December 5, 2024, https://aci.health.nsw.gov.au/projects/co-design/step-by-step.

[24] Australian Government Department of Health and Aged Care. National Lung Cancer Screening Program. 2025, accessed May 2, 2025, https://www.health.gov.au/our-work/nlcsp.

[25] K. L. A. Luo , K. Dunlop , M. McFadden , et al., *User Testing of Information Materials Developed for the Australian National Lung Cancer Screening Program: A Qualitative Study* (In press). 10.1111/hex.70592 PMC12891978 41668554

[26] K. L. A. Dunlop , D. Luo , et al., “Preferences for Baseline Key Messages for a National Lung Cancer Screening Program: A Qualitative Study With the Community and Health Workforce,” Manuscript in Preparation.

[27] K. McFadden , N. J. Harrison , S. Rose , et al. “As for stigma? … I don't think I'd be going to my next‐door neighbour saying ‘I'm going for lung cancer screening’”: a qualitative study of the potential impacts of stigma during lung cancer screening in Australia. *Manuscript submitted for publication*. Published online 2025. 10.1093/ntr/ntag141 42384945

[28] K. McFadden , B. Nickel , N. Houssami , N. M. Rankin , and R. H. Dodd , “Psychosocial Impacts Of, and Barriers To, Lung Cancer Screening: An International Qualitative Study of Multidisciplinary Health Professionals' Perspectives,” Patient Education and Counseling 137 (2025): 109172, 10.1016/J.PEC.2025.109172. 40378776

[29] A. L. Stangl , V. A. Earnshaw , C. H. Logie , et al., “The Health Stigma and Discrimination Framework: A Global, Crosscutting Framework to Inform Research, Intervention Development, and Policy on Health‐Related Stigmas,” BMC Medicine 17, no. 1 (2019): 31, 10.1186/s12916-019-1271-3. 30764826 PMC6376797

[30] S. Timmermans and I. Tavory Theory Construction in Qualitative Research. 2012;30(3):167–186, 10.1177/0735275112457914.

[31] J. Thompson , “A Guide To Abductive Thematic Analysis,” Qualitative Report 27, no. 5 (2022): 1410–1421, 10.46743/2160-3715/2022.5340.

[32] Australian Bureau of Statistics. *Remoteness Areas (Jul2021‐Jun2026)*.; 2023, accessed December 10, 2024, https://www.abs.gov.au/statistics/standards/australian-statistical-geography-standard-asgs-edition-3/jul2021-jun2026/remoteness-structure/remoteness-areas.

[33] L. S. Wallace , E. S. Rogers , S. E. Roskos , D. B. Holiday , and B. D. Weiss , “Brief Report: Screening Items to Identify Patients With Limited Health Literacy Skills,” Journal of General Internal Medicine 21, no. 8 (2006): 874–877, 10.1111/J.1525-1497.2006.00532.X. 16881950 PMC1831582

[34] M. Rigney , E. Rapsomaniki , L. Carter‐Harris , and J. C. King , “A 10‐Year Cross‐Sectional Analysis of Public, Oncologist, and Patient Attitudes About Lung Cancer and Associated Stigma,” Journal of Thoracic Oncology 16, no. 1 (2021): 151–155, 10.1016/J.JTHO.2020.09.011. 33011390 PMC8355604

[35] J. L. Studts , C. M. Deffendall , S. L. McCubbin , et al., “Examining Evidence of Lung Cancer Stigma Among Health‐Care Trainees,” JNCI Monographs 2024, no. 63 (2024): 20–29, 10.1093/JNCIMONOGRAPHS/LGAE010. PMC11151328 38836527

[36] Australian Government Department of Health and Aged Care. National Lung Cancer Screening Program information for healthcare providers | Australian Government Department of Health and Aged Care. 2025, accessed April 9, 2025, https://www.health.gov.au/our-work/nlcsp/for-healthcare-providers#workforce-education-and-information.

[37] H. Wilson , “How Stigmatising Language Affects People in Australia who use Tobacco, Alcohol and Other Drugs,” Australian Journal of General Practice 49, no. 3 (2020): 155–158, 10.31128/ajgp-07-19-4998. 32113210

[38] R. D. Goodwin and L. K. Walker , “Time to Stop Using the Word 'Smoker': Reflecting on the Role of Language in Advancing the Field of Nicotine and Tobacco Research,” Nicotine & Tobacco Research 24, no. 12 (2022): 1847–1848, 10.1093/ntr/ntac218. 36130319

[39] American Cancer Society National Lung Cancer Roundtable, *Lung Cancer Stigma Communications Assessment Tool (LCS‐CAT) Alternatives Suite* (2024), https://nlcrt.org/resource-center/.

[40] Global Lung Cancer Patient Council (Roche), *Promoting Good Mental Health in People With Lung Cancer* (2022), https://medically.roche.com/content/dam/sh/mental-health-in-lung-cancer/lungmental-health-leaflet.pdf.

[41] H. Pinnock , M. Barwick , C. R. Carpenter , et al., “Standards for Reporting Implementation Studies (Stari) Statement,” BMJ (Clinical research ed.) 356 (2017): 6795, 10.1136/BMJ.I6795. PMC5421438 28264797

[42] E. Proctor , H. Silmere , R. Raghavan , et al., “Outcomes for Implementation Research: Conceptual Distinctions, Measurement Challenges, and Research Agenda,” Administration and Policy in Mental Health and Mental Health Services Research 38, no. 2 (2011): 65–76, 10.1007/S10488-010-0319-7. 20957426 PMC3068522

[43] T. Wang , J. Y. Tan , X. L. Liu , and I. Zhao , “Barriers and Enablers to Implementing Clinical Practice Guidelines in Primary Care: An Overview of Systematic Reviews,” BMJ Open 13, no. 1 (2023): e062158, 10.1136/BMJOPEN-2022-062158. PMC9827241 36609329

[44] T. Reis , I. Faria , H. Serra , and M. Xavier , “Barriers and Facilitators to Implementing a Continuing Medical Education Intervention in a Primary Health Care Setting,” BMC Health Services Research 22, no. 1 (2022): 638, 10.1186/S12913-022-08019-W/TABLES/2. 35562695 PMC9099036

[45] D. S. Goldberg , “on Stigma & Health,” Journal of Law, Medicine & Ethics 45, no. 4 (2017): 475–483, 10.1177/1073110517750581.

[46] S. Zheng , S. Liu , Q. Yang , et al., “The Effectiveness of Interventions to Reduce Cancer‐Related Stigma: An Integrative Review,” Journal of Clinical Nursing 33, no. 7 (2024): 2438–2455, 10.1111/JOCN.17014. 38345136

[47] S. C. Banerjee , N. Haque , C. L. Bylund , et al., “Responding Empathically to Patients: A Communication Skills Training Module to Reduce Lung Cancer Stigma,” Translational Behavioral Medicine 11, no. 2 (2021): 613–618, 10.1093/TBM/IBAA011. 32080736 PMC7963287

[48] S. C. Banerjee , N. Haque , E. A. Schofield , et al., “Oncology Care Provider Training in Empathic Communication Skills to Reduce Lung Cancer Stigma,” Chest 159, no. 5 (2021): 2040–2049, 10.1016/J.CHEST.2020.11.024. 33338443 PMC8129726

[49] S. C. Banerjee , C. Asuzu , B. Mapayi , et al., “Feasibility, Acceptability, and Initial Efficacy of Empathic Communication Skills Training to Reduce Lung Cancer Stigma in Nigeria: A Pilot Study,” JNCI Monographs 2024, no. 63 (2024): 30–37, 10.1093/JNCIMONOGRAPHS/LGAE006. PMC12104141 38836528

[50] S. Wade , P. Ngo , Y. He , et al. Estimates of the eligible population for Australia's targeted National Lung Cancer Screening Program, 2025–2030 | PHRP, https://www.phrp.com.au/. 2024;35(1), 10.17061/PHRP34342410. 40443073

[51] E. Bilenduke , S. Anderson , A. Brenner , et al., “Equitable Implementation of Lung Cancer Screening: Avoiding Its Potential to Mirror Existing Inequities Among People Who Use Tobacco,” Cancer Causes & Control: CCC 34, no. 1 (2023): 209–216, 10.1007/S10552-023-01790-Z/METRICS. 37713024 PMC10689540

