# Supplementary material for: Reducing Stigma in Lung Cancer Screening: Co‐Design of a Targeted Resource for Health Professionals in Australia
Source: Health Expect. 2026 Apr 27;29(2):e70571. doi: 10.1111/hex.70571 (PMC13114764; doi:10.1111/hex.70571)
Supplement: Supplementary file 1 — Appendices HEX jan. [file HEX-29-e70571-s001.pdf]

APPENDIX A. Final co-designed resource

REDUCING STIGMA IN THE NATIONAL LUNG CANCER SCREENING PROGRAM

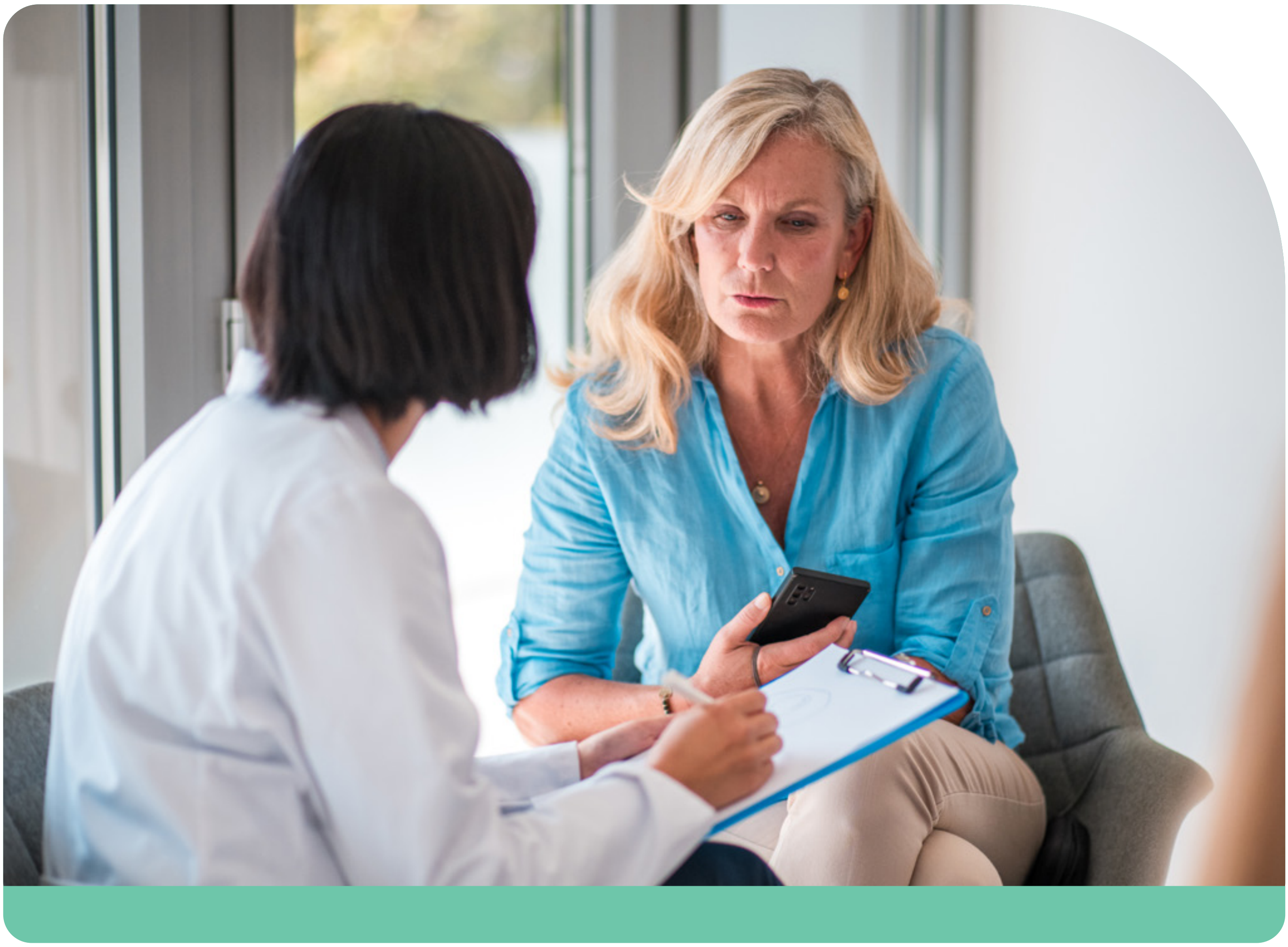

Participants eligible for the National Lung Cancer Screening Program will have a history of tobacco cigarette smoking. They have likely experienced stigma and discrimination because of this, and as a result may be hesitant about lung cancer screening.

It is critical to minimise stigma associated with smoking and cancer risk. This can help address barriers to a person participating in the program or seeking medical help more broadly.

Australian Government

NATIONAL LUNG CANCER SCREENING PROGRAM

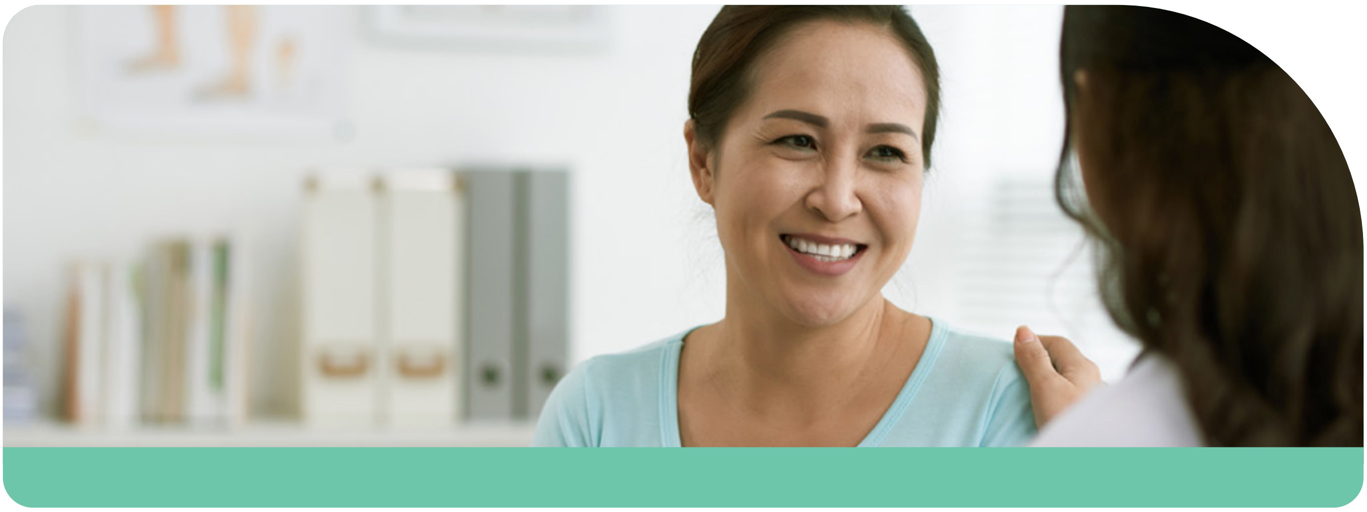

When talking about lung cancer screening, it is important to keep in mind that:

- People who smoke or have smoked often feel, or expect to feel, judged in healthcare encounters. In the past they may have felt discriminated against, not listened to, blamed, or that 'everything is brought back to smoking'.

- People may find talking about the eligibility criteria for lung cancer screening uncomfortable. They might be reluctant to discuss details, under-report smoking information and/or not want others to know about their smoking history or participation in screening.

- People who smoke often report feeling shame, guilt and embarrassment about their smoking history. They may feel that they should live with the consequences of smoking or that they don't deserve healthcare services like screening.

- Nicotine dependence is a clinical condition. Many people who smoke feel as if they have 'tried everything' to stop. If people have tried to quit in the past and started smoking again, they may feel a sense of failure or disappointment, which can impact their self-efficacy or motivation to try again.

- People who started smoking decades ago did not have the benefit of current tobacco control measures, research or education about the harms of tobacco smoking. Health warnings on cigarette packaging began in 1973, with nationally consistent health warnings only required in 1995.[1]

- Tobacco use is driven by harmful commercial interests and entrenched normalisation. In the past, industry marketing pushed smoking as 'cool' or 'glamorous', and in some cases as having health benefits. Australia only completely banned tobacco advertising in the 1990s,[1] and industry agendas still impact use.

- Smoking and its harms disproportionately impact Aboriginal and Torres Strait Islander peoples and communities.

Things you can do to minimise stigma when communicating about lung cancer screening:

✔ Remind people that they do not need to quit smoking to take part in the program.

✔ Frame smoking as a dependence on nicotine, not a lifestyle choice.

✔ Exercise empathy about the cultural, social and commercial determinants of someone's smoking history. Cigarettes were designed to be addictive.

✔ Encourage self-compassion around smoking:

- People are not perfect. Perceived flaws or failings (such as difficulty stopping or reducing tobacco use) are part of the human experience and are normal.
- Many people experience these feelings.
- Suggest that people try to give themselves patience, understanding and care – treat themselves like they would a good friend.
- Everyone deserves equal care, support and respect. Nobody deserves lung cancer.

✔ Remind people that screening is confidential and that others do not have to find out. However, having a social support network can be helpful during the screening process.

✔ Use strength-based messages to discuss screening and smoking cessation:

- Lung cancer screening may help to find lung cancer early when it is easier to treat, which can improve quality of life.
- It often takes multiple attempts to reduce or stop smoking entirely. Each try is a positive step, and a chance to learn more about what will work best for someone.
- There are many benefits of stopping or reducing smoking:

  Why Quit Smoking (www.health.gov.au/topics/smoking-vaping-and-tobacco/how-to-quit/why-quit-smoking)

  The health benefits of quitting smoking (www.quit.org.au/articles/the-health-benefits-of-quitting-smoking)

✔ There are a variety of supports available for people to stop or reduce smoking if they choose. These include tools and tips available through the **National Cessation Platform (quit.org.au)** and via the **MyQuitBuddy mobile app**

For healthcare providers, the **Quit Centre website** provides information, education and resources on smoking cessation to support patient care through cessation pathways.

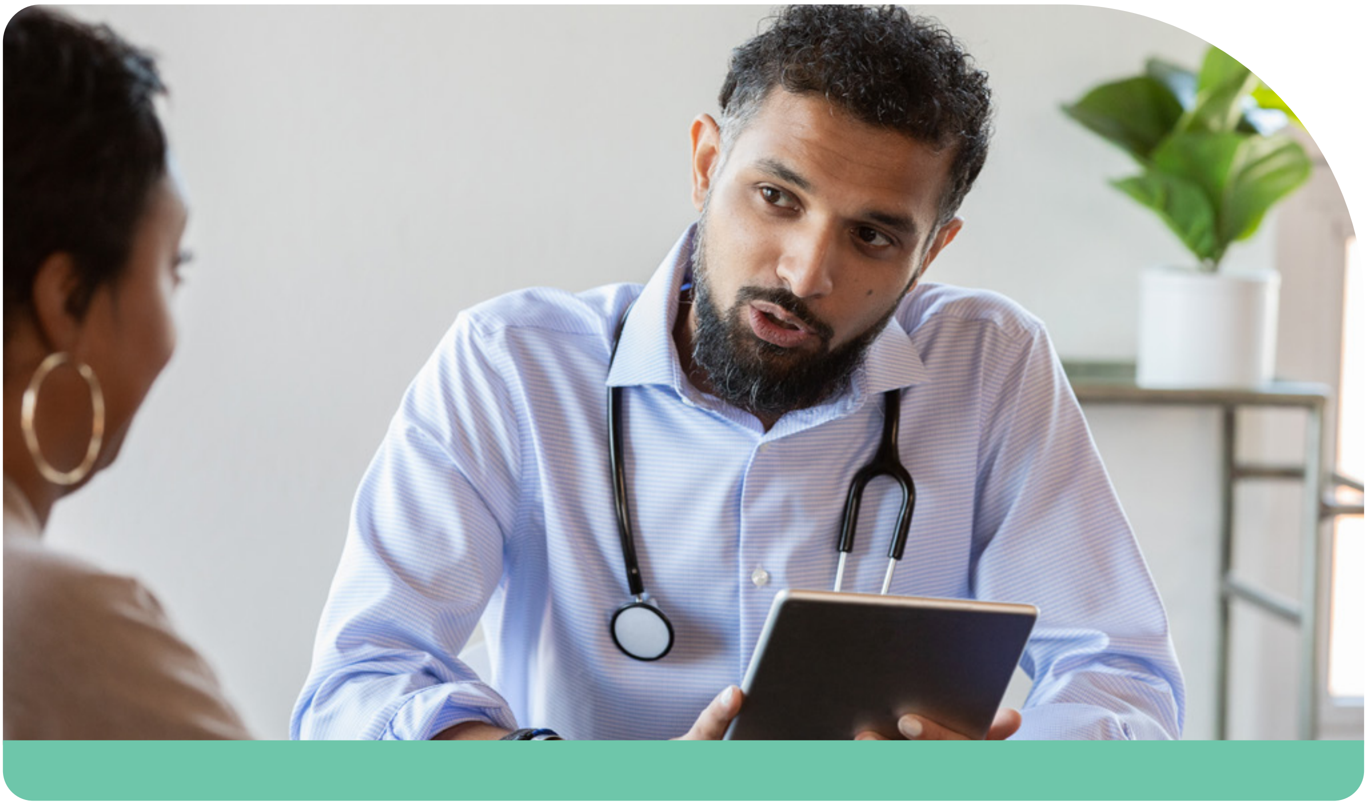

Lexicon guide for using person-first and empowerment language[2,3]

The words we use matter. Thoughtful use of language when communicating about lung cancer screening can help empower participants and reduce stigma.

Instead of ...		Use person-first and empowerment language
**Smoker**	→	Person who smokes
**Ex-smoker**	→	Person with a smoking history
**Nicotine addict**	→	Person with nicotine dependence
**Habit**	→	Dependence on nicotine
**Lifestyle**	→	Smoking or tobacco use behaviour
**Willpower**	→	Access to tools or resources to support stopping the use of tobacco
**Admitter or denier**	→	Someone with conflicting responses about smoking behaviour
**Willing/unwilling; non-compliant**	→	Unable, not able to; chooses not to

References:

1. Australian Government Department of Health and Aged Care. Tobacco control timeline. 2018. https://www1.health.gov.au/internet/publications/publishing.nsf/Content/tobacco-control-toc~timeline.

2. American Cancer Society National Lung Cancer Roundtable. Lung Cancer Stigma Communications Assessment Tool (LCS-CAT) Alternatives Suite 2024. https://nlcrt.org/resource-center.

3 . Global Lung Cancer Patient Council, Roche. Promoting good mental health in people with lung cancer 2022. https://medically.roche.com/content/dam/sh/mental-health-in-lung-cancer/ lung-mental-health-leaflet.pdf.

DT0004838_V01

www.health.gov.au/nlcsp

APPENDIX B. Mapping of key content themes onto final co-designed resource

KEY:

Theme 1: Taking the onus off the individual

Theme 2: Fostering understanding and empathy

Theme 3: Positive framing

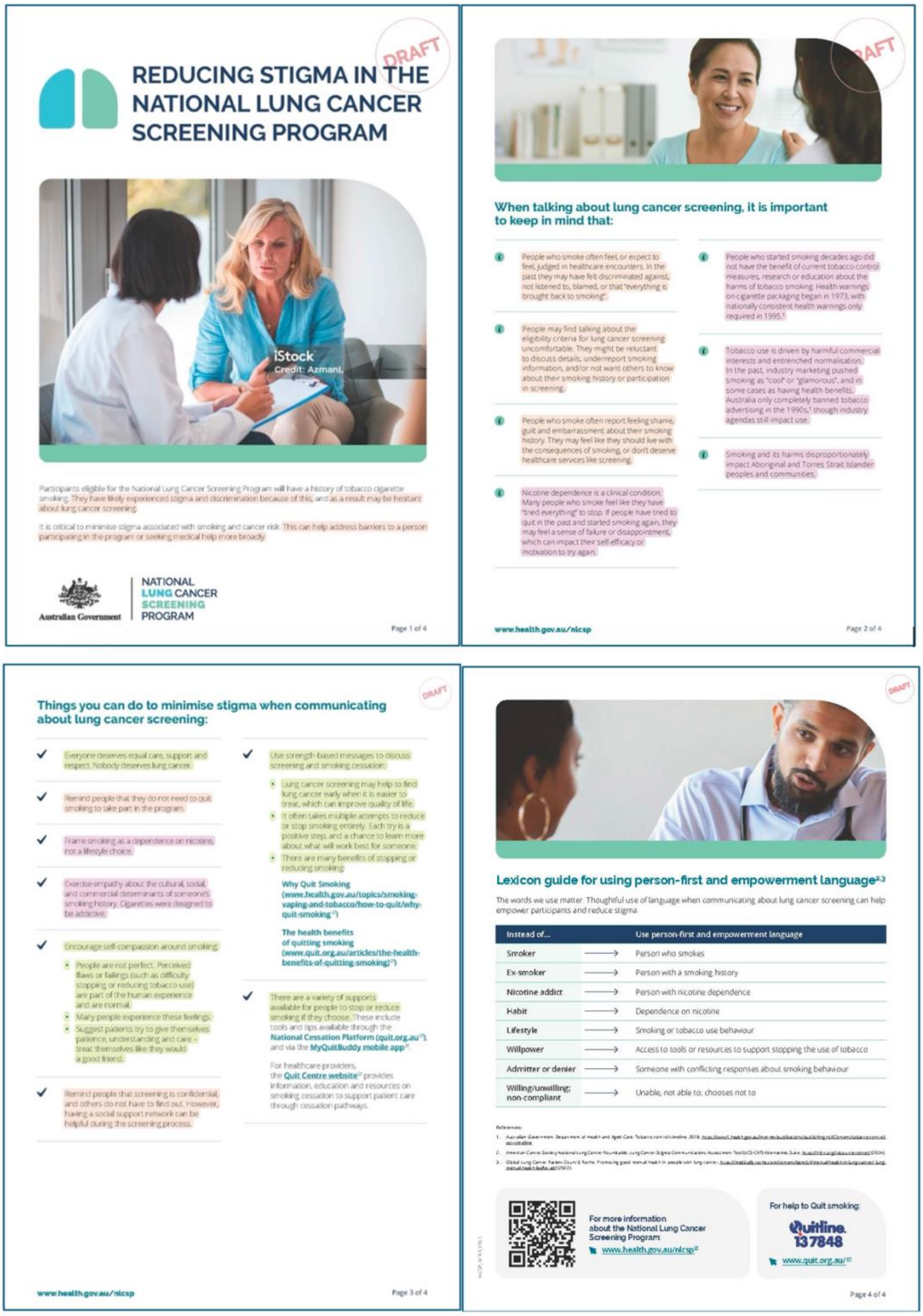

DRAFT

REDUCING STIGMA IN THE NATIONAL LUNG CANCER SCREENING PROGRAM

iStock
Credit: AzmanL

Participants eligible for the National Lung Cancer Screening Program will have a history of tobacco cigarette smoking. They have likely experienced stigma and discrimination because of this, and as a result may be hesitant about lung cancer screening.

It is critical to minimise stigma associated with smoking and cancer risk. This can help address barriers to a person participating in the program or seeking medical help more broadly.

Australian Government | NATIONAL LUNG CANCER SCREENING PROGRAM

Page 1 of 4

DRAFT

When talking about lung cancer screening, it is important to keep in mind that:

- People who smoke often feel, or expect to feel, judged in healthcare encounters. In the past they may have felt discriminated against, not listened to, blamed, or that "everything is brought back to smoking".
- People may find talking about the eligibility criteria for lung cancer screening uncomfortable. They might be reluctant to discuss details, underreport smoking information, and/or not want others to know about their smoking history or participation in screening.
- People who smoke often report feeling shame, guilt and embarrassment about their smoking history. They may feel like they should live with the consequences of smoking, or don't deserve healthcare services like screening.
- Nicotine dependence is a clinical condition. Many people who smoke feel like they have "tried everything" to stop. If people have tried to quit in the past and started smoking again, they may feel a sense of failure or disappointment, which can impact their self-efficacy or motivation to try again.
- People who started smoking decades ago did not have the benefit of current tobacco control measures, research or education about the harms of tobacco smoking. Health warnings on cigarette packaging began in 1973, with nationally consistent health warnings only required in 1995.[1]
- Tobacco use is driven by harmful commercial interests and entrenched normalisation. In the past, industry marketing pushed smoking as "cool" or "glamorous", and in some cases as having health benefits. Australia only completely banned tobacco advertising in the 1990s,[1] though industry agendas still impact use.
- Smoking and its harms disproportionately impact Aboriginal and Torres Strait Islander peoples and communities.

www.health.gov.au/nlcsp

Page 2 of 4

DRAFT

Things you can do to minimise stigma when communicating about lung cancer screening:

- ✓ Everyone deserves equal care, support and respect. Nobody deserves lung cancer.
- ✓ Remind people that they do not need to quit smoking to take part in the program.
- ✓ Frame smoking as a dependence on nicotine, not a lifestyle choice.
- ✓ Exercise empathy about the cultural, social, and commercial determinants of someone's smoking history. Cigarettes were designed to be addictive.
- ✓ Encourage self-compassion around smoking:
  - People are not perfect. Perceived flaws or failings (such as difficulty stopping or reducing tobacco use) are part of the human experience and are normal.
  - Many people experience these feelings.
  - Suggest patients try to give themselves patience, understanding and care – treat themselves like they would a good friend.
- ✓ Remind people that screening is confidential, and others do not have to find out. However, having a social support network can be helpful during the screening process.
- ✓ Use strength-based messages to discuss screening and smoking cessation:
  - Lung cancer screening may help to find lung cancer early when it is easier to treat, which can improve quality of life.
  - It often takes multiple attempts to reduce or stop smoking entirely. Each try is a positive step, and a chance to learn more about what will work best for someone.
  - There are many benefits of stopping or reducing smoking:

    Why Quit Smoking (www.health.gov.au/topics/smoking-vaping-and-tobacco/how-to-quit/why-quit-smoking)

    The health benefits of quitting smoking (www.quit.org.au/articles/the-health-benefits-of-quitting-smoking))
- ✓ There are a variety of supports available for people to stop or reduce smoking if they choose. These include tools and tips available through the **National Cessation Platform (quit.org.au)**, and via the **MyQuitBuddy mobile app**.

  For healthcare providers, the **Quit Centre website** provides information, education and resources on smoking cessation to support patient care through cessation pathways.

www.health.gov.au/nlcsp

Page 3 of 4

DRAFT

Lexicon guide for using person-first and empowerment language[2,3]

The words we use matter. Thoughtful use of language when communicating about lung cancer screening can help empower participants and reduce stigma.

Instead of...		Use person-first and empowerment language
Smoker	→	Person who smokes
Ex-smoker	→	Person with a smoking history
Nicotine addict	→	Person with nicotine dependence
Habit	→	Dependence on nicotine
Lifestyle	→	Smoking or tobacco use behaviour
Willpower	→	Access to tools or resources to support stopping the use of tobacco
Admitter or denier	→	Someone with conflicting responses about smoking behaviour
Willing/unwilling; non-compliant	→	Unable, not able to; chooses not to

References:

1. [illegible]
2. [illegible]
3. [illegible]

For more information about the National Lung Cancer Screening Program: www.health.gov.au/nlcsp

For help to Quit smoking: Quitline 137848 www.quit.org.au/

Page 4 of 4

APPENDIX C: Workshop activities and content

<table>
<tr><td>Workshop #</td><td>Topics</td><td>Time</td></tr>
<tr><td rowspan="7">Workshop 1
(2hr)</td><td>Introductions and rapport building
Acknowledgement of country
Workshop overview/timeline
Ground rules for discussion activities
Overview of co-design</td><td>15min</td></tr>
<tr><td>Overview of lung cancer screening
How Australia's National Lung Cancer Screening Program will work, including eligibility criteria</td><td>10min</td></tr>
<tr><td>What is stigma? What is discrimination? What might this look like in healthcare?[43]
Conceptualising stigma – different "levels" (intrapersonal, interpersonal and societal)[5]
ACTIVITY: What are some things people might feel stigmatised about in healthcare (e.g., smoking, mental health, weight, substance use or addiction, bias around age or sexuality)?[26] What are some potential impacts of stigma in healthcare (e.g., delayed help-seeking)?[43,44]
Exploration of stigmatising experiences in healthcare
Discussion of intersectional stigma
ACTIVITY: Stigma in lung cancer screening – What would this look like? What are the potential impacts?</td><td>30min</td></tr>
<tr><td>BREAK</td><td>5min</td></tr>
<tr><td>Summary of Session 1
Goal of co-design: People might experience stigma during lung cancer screening, including negative impacts like not wanting to get screened. We want to reduce or stop this by co-designing a strategy for the NLCSP to reduce stigma.</td><td>5min</td></tr>
<tr><td>ACTIVITY: We want to design a strategy for use in the NLCSP to reduce stigma.
What could this look like? – initial idea generation ("blue-sky thinking")
Content of strategy: What ideas and themes do we want to talk about? What is the messaging/wording?
Thematic examples used in smoking or lung cancer resources[44–47] (e.g., framing smoking as an addiction, not a lifestyle choice; tobacco industry influence)
Thematic examples used in stigma-reduction interventions in other contexts (e.g., impact of external factors)[48–50]
Types of messages (narrative/story-based, emotion-based)[49–53]
Delivery of strategy: Where is it delivered during lung cancer screening? How is it delivered?
Pathway/touchpoints of the NLCSP where a strategy/intervention may be implemented (e.g., as part of promotional materials, decision aids or educational interventions).</td><td>45min</td></tr>
<tr><td>Thank you, wrap-up & next steps</td><td>10min</td></tr>
<tr><td>Workshop 2
(1hr)</td><td>Acknowledgement of country
Workshop overview/timeline</td><td>10min</td></tr>
</table>

	Ground rules for discussion activities<br>Recap of Workshop 1:<br>Lung cancer screening and the Australian NLCSP<br>Stigma, discrimination and its impacts in lung cancer screening	
	ACTIVITY: Presentation, discussion and revision of developed resource<br>Overview of the developed resource (4 pages, aimed at health professionals in the program).<br>How the initial workshop and other consultation activities informed the design.<br>Content of resource: Presentation and discussion of key themes and content on each page.<br>Delivery of resource: Feedback on format and design of resource.	45min
	Thank you, wrap-up & next steps	5min
